# Emotional relevance and prejudice: testing the differentiated effect of incidental disgust on prejudice towards ethnic minorities

**DOI:** 10.3389/fpsyg.2023.1177263

**Published:** 2023-06-15

**Authors:** Emilia Pascal, Andrei Corneliu Holman, Felicia Mihaela Miluț

**Affiliations:** Department of Psychology, Faculty of Psychology and Education Sciences, Alexandru Ioan Cuza University, Iasi, Romania

**Keywords:** prejudice, incidental disgust, spill-over effect, ethnic minority, emotion relevance

## Abstract

Negative emotions such as disgust or anger influence the evaluation of minorities and amplify prejudice, stereotypes, and discrimination behaviors towards them. However, new discoveries suggest that these spillover effects might be more specific in the sense that the bias might occur only if the emotions are specific to the affect that is generally evoked by that particular minority, i.e. anger increases prejudice towards anger-relevant groups, and disgust towards disgust-relevant groups. Our study aimed to examine, the specificity of the spillover effects, namely the importance of emotion’s relevance to the prejudice towards out-groups. To test this hypothesis, we investigated the influence of incidental disgust on the evaluation of two minorities, one that is usually associated with disgust (the Roma minority) and one usually associated with anger (the Hungarian minority). We used a 2 × 2 between-subjects experimental design where we manipulated the emotion experienced by the participants (disgust versus neutral) and the target they evaluated (Romani or Hungarian minority). We tested the effects of these manipulations on three aspects of prejudice toward the target group: cognitive, affective, and behavioral. The results support the specificity of the spillover effect, by showing that incidental disgust increased prejudice only towards the disgust-relevant target, namely the Roma minority, and that the intensity of this emotion experienced by the participants mediates this effect. Moreover, incidental disgust increased not only the negative emotions associated with the Romani (i.e., the affective component) but also the negative cognitions associated with them and the desire to maintain an increased social distance (i.e., behavioral prejudice). These findings highlight the importance of emotions’ relevance in bias toward minorities and provide a starting point for future anti-discrimination interventions.

## 1. Introduction

It has been almost a century since emotions have been linked to prejudice and intergroup relations through the classical theory of frustration-aggression (Dollard et al., 1939). Although the investigation of stereotypes has been dominated for a significant period by the cognitive perspective, centered almost exclusively on attitudes (Allport, 1954), the focus tended to shift towards an emotional approach, as limitations of the cognitive perspective became evident (Taylor, 2007).

More recent theories approach prejudice in a more fine-grained manner by separating it into three components: cognitive, emotional, and behavioral (Kite et al., 2022). Stereotypes, the cognitive component, refer to beliefs about a person or a group, prejudice to the emotions one feels about them, and discrimination to the actual behaviors or intentions to behave in a certain way (Tejada et al., 2011). The present paper focuses on all three aspects of prejudice, aiming to examine specific pathways by which certain negative emotions bias the judgments of some minority groups while having no impact on the evaluations of others.

In Romania, the largest minority groups are represented by the Hungarian and Romani communities and the official numbers state that they represent 6.1%, respectively 3.4% of the population. However, it seems that these figures do not necessarily reflect reality. For example, Surdu (2019) states that many of the Roma ethnics are undocumented and a great majority refuse to declare their ethnic origins because they fear discrimination or unfair treatment. Moreover, another study (Surdu, 2016) concludes that the lack of an objective image of the Roma population size is an effect of certain political interests. In these conditions, it is almost impossible to know the real number of Romani ethnicities in Romania, however, we can confidently assume that it is greater than the official statistics state.

In general, the Romani minority has a negative public image, that is, at least in part, related to their poor living conditions (Anthonj et al., 2020). Most of the Roma population lives in old houses with improper sanitary conditions (Davis and Ryan, 2017). For example, in Romania more than 80% lack access to improved sanitation (an indoor toilet) or water sources (Powell Doherty et al., 2018). As a recent meta-analysis points out (Anthonj et al., 2020), discrimination is both a cause and an effect of their restricted access to basic facilities such as running water, sanitation, and hygiene – WASH (Filčák et al., 2018). Roma ethnics are negatively perceived by the majority because they mostly live in segregated areas lacking WASH facilities, but the negative perceptions of the majority represent in themselves one of the reasons for the poor conditions they live in. In order to be able to improve the quality of their living conditions they need financial stability, which is dependent on their access to superior, better-paying jobs. However, in reality, that is often difficult as studies show that Romani are discriminated against in the job application process, and many employers still refuse to hire them (Messing and Bereményi, 2017). Another example of a similar Vicious circle starts in childhood with the fact that Roma ethnics are segregated and discriminated against in schools. The fact that they are not able to benefit from the same education opportunities as the majority, and often drop out because of bad treatment, negatively influences their chances of finding highly-paid jobs in the future (O’Higgins and Brüggemann, 2014).

Moreover, empirical studies in Romania seem to support the fact that their improper living conditions represent a complex cause of discrimination. For example, Romanian doctors perceive the Roma minorities as dirty and lacking personal hygiene, which is one of the main reasons that incline them to avoid treating Roma patients, thus hindering their access to medical services (Roman et al., 2013).

Recent empirical studies confirm that associating disgust with the Roma minority is not specific only to the Romanian population, but was also discovered in Greek (Asimopoulos et al., 2019), Czech (Creţan et al., 2022) or Slovakian samples (Petrík et al., 2021). Moreover, other studies suggest that such negative emotions amplify the negative attitudes of the majority. For example, in an experimental study (Dalsklev and Kunst, 2015), it was found that reading a fictitious newspaper article about the low hygiene standards of the Roma minority elicited feelings of disgust that led to a more positive attitude towards deportation.

The Hungarian minority, on the other hand, is perceived differently, and the disputes between it and the majority seem to be related to other causes, namely the political context (Aluas and Matei, 1998). For more than three decades, the Hungarian minority pressured the Romanian government to adopt favorable laws related to the recognition of their rights and the preservation of their customs and language. Their efforts started to pay off in 1996 and since then much favorable legislation, such as Act 125 which allowed bilingual inscriptions in areas where minorities represented more than 20% of the population (Act No. 125 on Local Administration, 2001), has been adopted. Moreover, a recent study Veres (2015) points out that only 11% of the individuals from the Hungarian minority consider themselves Romanian citizens first, while the majority prefer to define themselves as a separate ingroup, thus creating more conflicts between them and the majority.

Contrary to the Roma population, the Hungarian minority is not perceived as inferior, quite the contrary, it is perceived as a powerful group that could pose a threat to the territorial integrity of the state (Aluas and Matei, 1998). In this particular context, the relationship between the majority and the Hungarian minority mostly generates emotions of fear and resentment (Daftary and Grin, 2003), while the interactions between Romanians and the Roma minority often elicit mostly emotions of disgust and a tendency towards rejection (Hajioff, 2000).

The bidirectional link between emotions and prejudice has been proven by numerous studies. On one hand, different outgroups elicit different prejudice-related emotions (Tapias et al., 2007), but on the other hand, the emotions themselves can influence the perception of a certain group. This spill-over effect, namely the transfer of the negative valence associated with a certain emotion to the evaluation of the target, has emerged even when there is no relation between the activation of the affect itself and the group that is being judged, as in the case of incidental emotions (Paolini et al., 2021).

Out of all negative emotions, disgust is probably the most frequently researched in relation to stereotyping outgroups, and results have systematically shown its negative biasing influences (Taylor, 2007). For example, some studies (Vartanian et al., 2016) showed that the disgust elicited by people with obesity amplified participants’ stereotypes and the desire to maintain social distance. Other researchers have shown that disgust-related words are intentionally used in anti-group texts because they induce negative attitudes (Taylor, 2007). Finally, other studies (Kiss et al., 2020) found that even personal factors, such as individual differences related to sensibility to disgust, are associated with more negative attitudes towards minorities.

The main goal of our study is to further investigate the negative effect of disgust on prejudice towards two different minority groups, i.e., Roma and Hungarian, in a sample of Romanian participants. While most previous studies (e.g., Dalsklev and Kunst, 2015) examined the effects of integral affective states on the target groups, we aim to test the influence of incidental emotions. In the aforementioned study, disgust was induced through a fictitious newspaper article about the poor hygiene of the Roma ethnics. One can argue that the observed effect was not only determined by the emotion experienced alone, but also by the depreciative information concerning the target group. By using unrelated emotion–inducing materials that elicit incidental emotions we can study the specific effects of the emotion itself. Moreover, our study aims to compare the effects of incidental disgust on the evaluation of the two targets (the Roma and the Hungarian minority). This would empirically test two competitive explanatory models: the overall spill-over effect of negative emotions which refers to the idea that negative emotions determine harsher evaluations, irrespective of their relevance to the target (Vartanian et al., 2016) and the specificity of the spill-over effects which states that, in order for the biasing effect to occur, the emotion induced needs to be specific to the target evaluated (Dasgupta et al., 2009; Paolini et al., 2021).

The first model is based on several research findings (e.g., Taylor, 2007; Vartanian et al., 2016) indicating that incidental disgust has spill-over effects increasing prejudice towards minorities, thus biasing their evaluation in an apparently global manner. Yet, other studies (DeSteno et al., 2004; Dasgupta et al., 2009; Paolini et al., 2021) suggest that this effect is more specific. For example, in one study (Dasgupta et al., 2009), researchers tested the effect of three negative incidental emotions (anger, disgust, and sadness) on prejudice. Their results showed that anger and disgust amplified the negative attitudes toward different groups, but in specific ways. Anger amplified the prejudice only towards anger-relevant minorities (Arab men), while disgust only towards disgust-relevant groups (homosexual men). In other words, according to their findings, negative emotions can amplify negative judgments only if they are specific to the emotion typically elicited by the target group. A more recent research (Paolini et al., 2021) confirms the negative effects of incidental anger on prejudice towards Arabic men, using a different procedure (i.e., visualization scenarios), which further emphasizes the importance of the congruence between the induced incidental emotion and the one typically elicited by the target out-group.

In our study, we aim to further test the specificity of the spill-over effect by examining whether this effect is also instilled by another incidental negative emotion (i.e., disgust), and with other prejudiced minorities. In this respect, while previous studies (Dasgupta et al., 2009) compared two different types of minorities (a sexual and an ethnic minority), we aim to investigate the specificity of the spill-over effect on the prejudice towards two ethnic minorities: the Roma and the Hungarian. The two minorities typically elicit different negative emotions in members of the Romanian majority: the Romani ethnics are associated with disgust (Asimopoulos et al., 2019; Petrík et al., 2021; Creţan et al., 2022), while the Hungarian minority generally elicits fear and resentment (Daftary and Grin, 2003). This provides an appropriate setting for testing the emotion–related specificity of the spill-over effects. As disgust is the emotion induced in our experimental design, this manipulation should amplify only the prejudice towards the Roma population, while having no significant effects on the evaluation of the individuals from the Hungarian minority.

## 2. Method

### 2.1. Participants

Two hundred and twelve participants (*N_men_* = 62) took part in our study. The majority were undergraduate students (*N*_undergraduate_ = 112) from a wide range of universities, aged 18 to 63 years old (*M* = 24.55, SD = 8.24). They were recruited mostly through social media platforms such as Facebook or Twitter. We used a 2 × 2 between-subjects design where we manipulated the emotion felt by the participants (disgust versus neutral) and the target they evaluated (Romani or Hungarian). All participants identified as Romanians and had normal, or corrected to normal vision.

### 2.2. Procedure

The participants completed the questionnaire online via the Qualtrics platform. Upon reading the informed consent, and agreeing to take part in our research, they were randomly assigned to one of the four experimental conditions (elicited emotion: disgust vs. neutral X target: Romani vs. Hungarian target).

Participants were exposed for 15 s to either a neutral or a disgusting picture (participants were unable to skip until the time had passed). After viewing the picture, participants completed the manipulation check task, then they were asked to think about the Romani or the Hungarian minority and complete the Prejudiced Attitude Tricomponent Test. To avoid all ambiguities, participants were reminded, at the beginning of each scale of this measure, of the minority to which they were required to refer to while completing the items.

### 2.3. Materials and measures

#### 2.3.1. Prejudice

Prejudice was measured with the Prejudiced Attitude Tricomponent Test (Tejada et al., 2011). The scale measures all three components of prejudice: cognitive, emotional, and behavioral, and the results of this study support the differentiation between the three facets and the validity of the instrument. The answers to the items specific to each scale were added to compute an overall score for each component (Mañas et al., 2012).

The cognitive component scale (*α* = 0,87) consists of eight items, evaluated on a 5-point Likert scale (from 1 – Very bad to 5 – Very good) that assessed various cognitive aspects related to the out-group. More specifically, the scale measured beliefs related to various socio-cultural areas such as politics, social welfare, employment, economy, social family, religion, and values. All scores were reversed so that higher overall scores reflected a more prejudicial judgment.

The affective component of the scale (*α* = 0,76), evaluated on a 5-point Likert scale (from 1 – Very bad to 5 – Very good), measures 7 “subtle” emotions related to the out-group evaluation. Three of the emotions assessed were positive (admiration, friendliness, and respect) and four were negative (distrust, discomfort, insecurity, and indifference). The scores for positive emotions were reversed so that higher overall scores reflected more prejudice-related emotions.

The behavioral component of the scale measures the relation (distant or close) participants are willing to have with members of the target group. The scale consists of one item, based on the Social Distance Scale (Bogardus, 1959), that requires participants to choose one of the five types of relations. According to their choices, participants scores ranged from 5, which corresponded to a very distant relation (“not having any relations with people in the out-group”) to 0, a very close relation (“form a family with a person from the out-group”).

#### 2.3.2. Emotion manipulation

The emotion-inducing task consisted in presenting participants for 15 s with one of two stimuli: a neutral image (a piece of white furniture with a small plant on it) or a disgusting picture (an image of a dirty closet). The procedure is similar to that used in other studies and has been proven efficient in inducing disgust (e.g., Moretti and di Pellegrino, 2010; Clifford and Wendell, 2016). Moreover, a recent review (Siedlecka and Denson, 2019) suggested that visual stimuli represent the most effective material in inducing disgust.

#### 2.3.3. Manipulation check

The effectiveness of the manipulation was assessed using one item asking participants to evaluate, on a 5-point Likert scale (1- not at all, and 5- very much) to what degree they feel each of the six basic emotions (sadness, happiness, surprise, fear, anger, and disgust). The emotions were chosen based on a previous study (Sarlo et al., 2005) where researchers used this procedure to evaluate the effects of a disgusting material, and its results support the validity of this measure.

## 3. Results

### 3.1. Manipulation checks

We analyzed the effects of our experimental manipulation of emotion on affective ratings using the Independent Samples t-test. Results indicate that participants in the disgust condition reported more intense disgust (*M* = 4.38, SD = 0.96) than those in the neutral condition (*M* = 1.21, SD = 0.56; *t*(210) = 29.76; *p* < 0.001). We also found other emotional effects of the experimental manipulation, as participants in the disgust condition reported being more surprised (*M* = 3.23, SD = 1.32 vs. *M* = 1.59, SD = 0.87; *t*(210) = 10.74; *p* < 0.001) and angrier (*M* = 2.36, SD = 1.47 vs. *M* = 1.31, SD = 0.72; *t*(210) = 6.70; *p* < 0.001). Yet, the comparisons among the intensity of the six emotions reported by the participants in the experimental group indicate that the intensity of their experienced disgust was above that of all the other emotions (all *p*s < 0.01). There were no other significant differences between the two groups in their emotion ratings (all *p*s > 0.05).

### 3.2. Relationships between the three components of prejudice and the socio-demographic variables

The descriptive statistics and the relationships between the three components of prejudice measured by the Prejudiced Attitude Tricomponent Test and the socio-demographic variables are presented in Table 1. Results show positive associations between the three components of prejudice, while no relationships between these scales and gender, age, or education emerged as significant.

**Table 1:** Descriptive statistics and correlations between the three components of prejudice and the socio-demographic variables.

Variables	Min	Max	*M*	SD	1	2	3	4	5
1. Cognitive component	1	5	3.20	0.72	–				
2. Affective component	1.14	5	2.85	0.74	0.61^**^	–			
3. Behavioral component	0	5	1.69	1.73	0.57^**^	0.60^**^	–		
4. Gender	150 (78.8%) women				−0.04	−0.07	−0.03	–	
5. Age	18	63	24.71	8,25	−0.10	0.03	0.00	−0.12	–
6. Education	100 (47.2%) university graduates				−0.04	0.05	0.07	−0.10	0.50^**^

^**^p < 0.01; *N* = 212. All gender and education correlations represent point biserial coefficients.

### 3.3. Effects of the manipulated variables on the three components of prejudice

Next, we examined the effect of the two variables we manipulated (i.e., emotion and target evaluation) on each of the three components of prejudice using between-subjects analyses of variance (ANOVA). On the cognitive component of prejudice, we found a significant main effect of the emotional induction [*F*(1, 208) = 38.15, *p* < 0.001], participants in the disgust condition scoring higher on this scale than those in the neutral condition (*M* = 3.46, SD = 0.64 vs. *M* = 2.93, SD = 0.70). There was also a significant main effect of the ethnicity of the target evaluated [*F*(1,208) = 4.83, *p = 0*.03], participants evaluating Romani ethnics expressing stronger cognitive prejudice than those evaluating the individuals from the Hungarian minority (*M* = 3.27, SD = 0.80 vs. *M* = 3.10, SD = 0.63). We also found a significant interaction between the two independent variables [*F*(1,208) = 22.91, *p* < 0.001]. When exploring this interaction, we found that among participants evaluating the Romani target, those in the disgust condition expressed stronger cognitive prejudice than those in the emotionally neutral condition [*F*(1,103) = 58.81, *p* < 0.001; *M* = 3.77, SD = 0.57 vs. *M* = 2.81, SD = 0.70], but the effect of the emotional induction was not significant in the group evaluating Hungarian ethnics [*F*(1,105) = 0.99, *p* = 0.32; *M* = 3.17, SD = 0.56 vs. *M* = 3.04, SD = 0.69].

On the affective component of prejudice, results indicated a significant main effect of the emotional induction [*F*(1, 208) = 7,32, *p* = 0.007], participants in the disgust condition expressed stronger affective prejudice than those in the neutral condition (*M* = 2.96, SD = 0.81 vs. *M* = 2.72, SD = 0.66). We also found a significant main effect of the target evaluated [*F*(1, 208) = 17.07, *p* < 0.001], participants evaluating Romani ethnics scoring higher on this scale than those evaluating Hungarian ethnics (*M* = 3.02, SD = 0.79 vs. *M* = 2.66, SD = 0.65). The interaction between the two independent variables was also significant [*F*(1, 208) = 26.59, *p* < 0.001]. Participants in the disgust condition expressed stronger cognitive prejudice than those in the emotionally neutral condition concerning Roma ethnics [*F*(1,103) = 28.08, *p* < 0.001; *M* = 3.40, SD = 0.73 vs. *M* = 2.67, SD = 0.68], but the effect of the emotional induction was not significant among participants evaluating the individuals from the Hungarian minority [*F*(1,105) = 3.33, *p* = 0.07; *M* = 2.54, SD = 0.65 vs. *M* = 2.76, SD = 0.64].

**Figure 1:**
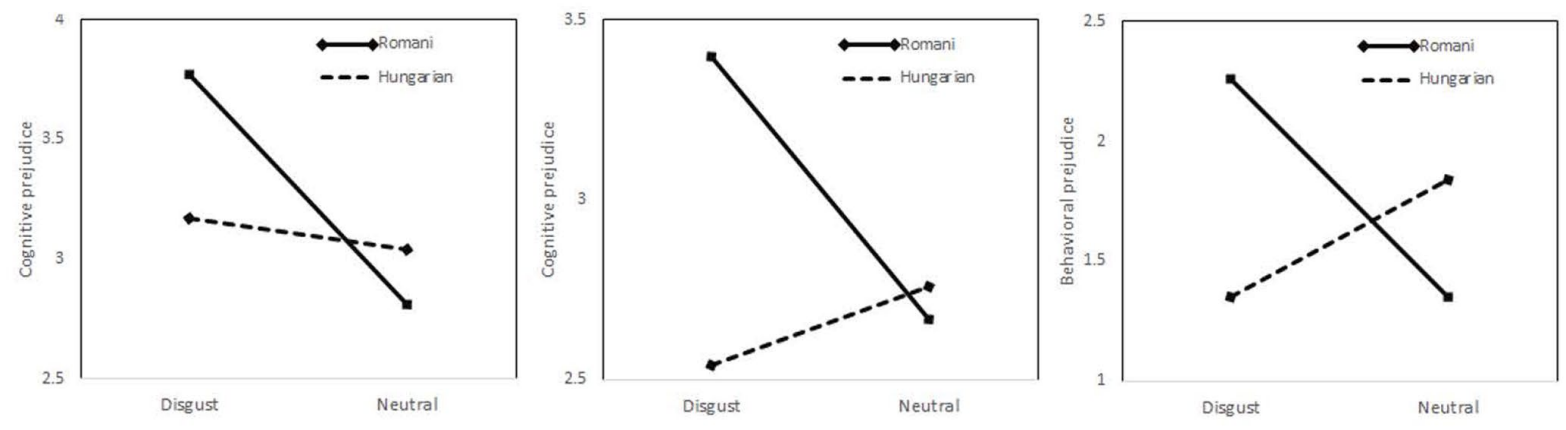
The interactions between emotional induction and ethnicity of the target evaluated on the intensity of three facets of prejudice.

On the behavioral component of prejudice, neither the emotional induction [*F*(1, 208) = 0.84, *p* = 0.36] nor the target evaluated [*F*(1, 208) = 0.78, *p* = 0.38] had significant main effects, but their interaction emerged as significant [*F*(1, 208) = 8.95, *p* = 0.003]. Among participants evaluating the Romani minority, those in the disgust condition expressed stronger behavioral prejudice than those in the emotional neutral condition [*F*(1,103) = 7.18, *p* = 0.009; *M* = 2.26, SD = 1.90 vs. *M* = 1.35, SD = 1.59], but the effect of the emotional induction was not significant in the group evaluating Hungarian ethnics [*F*(1,105) = 2.92, *p* = 0.13; *M* = 1.35, SD = 1.54 vs. *M* = 1.84, SD = 1.77]. The interactions between emotional induction and ethnicity of the target evaluated on the intensity of each of the three facets of prejudice are represented in Figure 1.

### 3.4. Experienced disgust as mediator and ethnicity of the target as moderator of the effects of emotional induction

**Figure 2:**
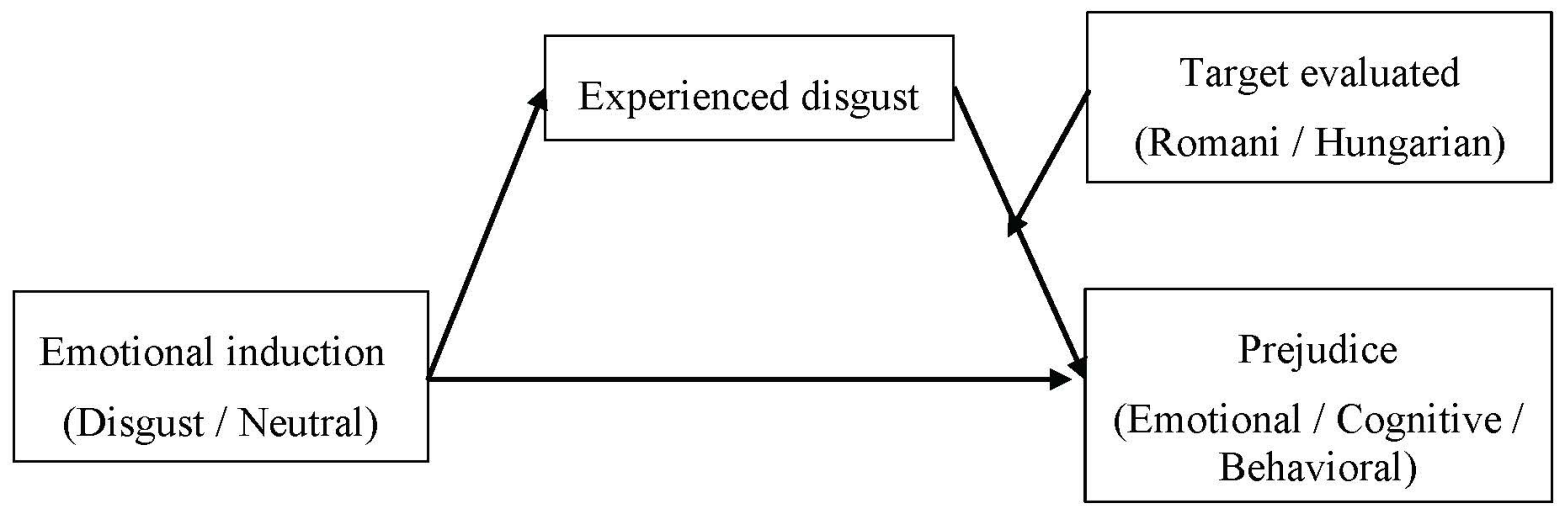
The hypothesized mediation model between emotional induction, disgust, and the three components of prejudice moderated by target ethnicity.

The results presented above indicate that the experimental manipulation of emotion increased all three facets of prejudice expressed, especially concerning the Romani minority. Next, we investigated whether these effects are mediated by the intensity of disgust experienced by the participants and whether this indirect effect varies according to the ethnicity of the target evaluated. The analyses were performed using Model 14 in PROCESS v. 4.1 macro for SPSS (Hayes, 2022) on 5,000 bootstrap samples, using a percentile bootstrapping approach for creating 95% confidence intervals. The hypothesized moderated mediation model in Figure 2 was tested using as dependent variable (Y) each of the three components of prejudice. In all three analyses, we introduced the manipulation of emotion as the independent variable (X), disgust as the mediator (M), and target ethnicity (W) as the moderator of the effect of X on M. The two affective states that emerged in the manipulation checks reported above as being significantly influenced by our emotional induction, besides disgust (i.e., surprise and anger) were introduced as covariates in all three models.

#### 3.4.1. The cognitive component

The results of the analysis of the model including the cognitive component of prejudice as the dependent variable confirmed the effect of our experimental manipulation on experienced disgust (*b* = 2.80, *p* < 0.001, CI: 2.56, 3.04). We also found that the effect of disgust on cognitive prejudice was moderated by target ethnicity, as the interaction between these variables emerged as a significant predictor (*b* = 0.22, *p* < 0.001, CI: 0.12, 0.32). Moreover, the overall mediation model of relationships between the emotional induction, the experienced disgust and cognitive prejudice was significantly moderated by target ethnicity, as indicated by the *index of moderated mediation* = 0.62, CI: 0.35, 0.91. The fact that the bootstrapped CI of this index did not include zero suggests that the indirect effect in this model (i.e., between the emotional induction, disgust, and cognitive prejudice) differ across the two ethnic groups evaluated by our participants (Hayes, 2015). Specifically, we found that this conditional indirect effect was significant in the group evaluating the Romani target (*B* = 0.53, CI: 0.22, 0.87), but not in the group evaluating the Hungarian target (*B* = −0.09, CI: −0.45, 0.28). This difference stemmed from the fact that, in the first group, the experienced disgust was a significant factor of cognitive prejudice (*b* = 0.19, *p* = 0.005, CI: 0.06, 0.32), and thus a significant mediator of the relationship between the emotional induction and cognitive prejudice, while in the group evaluating the Hungarian target disgust did not predict cognitive prejudice (*b* = −0.03, *p* = 0.66, CI: −0.17, 0.11).

#### 3.4.2. The affective component

Results indicated target ethnicity as a moderator of the effect of experienced disgust on affective prejudice (*b* = 0.27, *p < 0*.001, CI: 0.17, 0.38). The index of moderated mediation = 0.76, CI: 0.47, 1.06 also suggested that the conditional indirect effects of the emotional induction on affective prejudice via experienced disgust differed across the two target ethnic groups. The conditional indirect effect emerged as significant in participants who evaluated the Romani target (*B* = 0.89, CI: 0.51, 1.31), where experienced disgust was a significant factor of affective prejudice (*b* = 0.32, *p < 0*.001, CI: 0.18, 0.45), and thus mediated the effect of the emotional induction on affective prejudice. In the group evaluating the Hungarian target, the conditional indirect effect was not significant (*B* = −0.13, CI: −0.27, 0.52), as disgust was not significantly associated with affective prejudice (*b* = 0.05, *p* = 0.54, CI: −0.10, 0.19).

#### 3.4.3. The behavioral component

Target ethnicity was also a significant moderator of the effect of experienced disgust on behavioral prejudice (*b* = 0.40, *p = 0*.003, CI: 0.14, 0.67), as well as of the mediation relationships between the emotional induction, disgust, and this type of prejudice (index of moderated mediation = 1.13, CI: 0.42, 1.87). This conditional indirect effect was significant in the group evaluating the Romani target (*B* = 1.21, CI: 0.25, 2.28), but not in participants evaluating the Hungarian target (*B* = 0.08, CI: −0.96, 1.14). As in the cases of the other two components of prejudice, experienced disgust had a significant effect on behavioral prejudice in participants evaluating the Romani target (*b* = 0.43, *p = 0*.01, CI: 0.08, 0.79), but not in the Hungarian target condition (*b* = 0.03, *p* = 0.87, CI: −0.34, 0.40).

## 4. Discussion

The main aim of the present study was to investigate the extent of the emotional spill-over from incidental disgust to prejudice towards two of the most numerous minorities in Romania (i.e., Romany and Hungarian), varying in the emotion that the members of the majority typically associate them with. By experimentally manipulating the incidental emotion experienced by participants, we measured the influence of disgust on the three components of prejudice, i.e., emotional, cognitive, and behavioral, towards each of the two minority groups. Our results indicated that the spill-over effect from disgust to prejudice was target-specific across all three components, as disgust increased only the prejudicial reactions towards the Roma minority, which is typically associated with disgust in members of the majority (Hajioff, 2000; Roman et al., 2013; Dalsklev and Kunst, 2015).

A considerable number of studies showed that disgust has negative spill-over effects on prejudice towards different types of groups (Dalsklev and Kunst, 2015): some usually associated with disgust, such as obese people (Vartanian et al., 2016), or homosexual minorities (Kiss et al., 2020) but also on neutral out-groups, such as fictitious communities (Hodson et al., 2013). Our results, however, suggest that, at least in relation to ethnic minorities, the effect of negative emotions is more specific, amplifying prejudice only if it was relevant to the target evaluated. In our study, incidental disgust increased prejudice only towards the Roma minority, while having no effect on the evaluation of the Hungarian minority, typically associated with other negative feelings, i.e., anger and resentment (Daftary and Grin, 2003). Moreover, this specific spill-over effect from disgust to ethnic prejudice was observed in all three aspects of prejudice: cognitive, emotional, and behavioral.

Previous research has suggested that emotional influences on prejudice are specific. For example, DeSteno et al. (2004) showed that anger, and not sadness determined negative evaluations towards out-groups, and Dasgupta et al. (2009) found that anger increased prejudice only toward minorities that commonly evoke similar emotions (i.e., Arab men). Similarly, disgust did not affect the evaluation of anger-relevant minorities, but increased prejudice towards homosexual men, a minority usually associated with lack of purity. On the same target minority of Arabic men, Paolini et al. (2021) have replicated this pattern of effects, showing that for biasing effects to occur, the incidental emotion induced has to be relevant to the outgroup being evaluated.

Our research extends and confirms these findings related to the importance of the relevance of emotion towards the target, firstly by showing that this effect is not specific only to anger, as previously highlighted, but also occurs in the case of disgust. Secondly, the mediation analysis results provide additional support for the specific spillover hypothesis by showing that the intensity of disgust experienced is a significant predictor of the magnitude of prejudicial bias towards the Roma minority. Thirdly, our results suggest that incidental disgust does not affect only the emotional component of prejudice, but also biases the behavioral and cognitive evaluations. Participants who experienced disgust also reported more negative emotions towards Roma, such as anger or fear, and less positive emotions, like admiration. They also expressed an increased desire to maintain social distance and avoid direct interactions with members of this minority. Fourthly, disgust also increased the probability that participants would consider negative stereotypes about Roma’s work ethic and personal habits as true.

Lastly, our study also contributes to the existing knowledge by revealing the emotion-specific spill-over effect when comparing two different ethnic minorities. Past studies have compared different types of minorities (e.g., for example, an ethnic with a sexual minority) (Dasgupta et al., 2009) or included only the Arabic minority as the target group (Paolini et al., 2021). Furthermore, Paolini et al.’s (2021) study, which also highlighted the importance of the relevance of the incidental emotion to the target outgroup, used visualization tasks in order to induce emotions, a technique with limited efficacy for some basic emotions (Siedlecka and Denson, 2019). By using visual stimuli, which past research indicated to be the most efficient disgust-eliciting technique (Siedlecka and Denson, 2019), it is possible that the participants in the experimental group of our study experienced more intense emotional states.

The Roma community is generally perceived as a low-social-status minority, with lower levels of education (Van Cleemput et al., 2007). They usually live on the periphery of cities in poor conditions, without access to WASH and basic facilities to maintain proper hygiene (Davis and Ryan, 2017). In the media, they are also portrayed negatively (Kóczé and Rövid, 2017), as problematic parasite communities, that break the law and represent a social problem rather than as a vulnerable minority (van Baar, 2011).

Disgust is elicited by people that transgress moral norms (Chapman and Anderson, 2012), are dirty, or carry contagious diseases (Curtis, 2007). In Romania, the Roma minority is associated with all three of these aspects, thereby disgust is the emotion most commonly elicited by them in members of the majority (Creţan et al., 2022). This highlights their vulnerable status in Romanian society, as such negative emotions further strengthen the depreciative stereotypes and discrimination towards the Roma minority (e.g., Roman et al., 2013). Furthermore, this disgust–fueled prejudice against them is also difficult to tackle and reduce, as indicated by the observation that the interventions aiming to reduce discrimination towards Roma ethnics in Romania have often failed (Pascal, 2020). One possible reason could be related to the fact that they did not address the cause of the prejudice, namely the specific negative emotions elicited by this minority in members of the majority. Different minorities are associated with specific negative emotions (Madon et al., 2001; Tapias et al., 2007) and distinct emotions promote particular reactions. For example, anger triggers aggressive, confrontational behavior, while disgust quite the opposite, leads to avoidant reactions (Cottrell and Neuberg, 2005). By showing that the intensity of disgust increases all three aspects of prejudice (i.e., emotional, cognitive, and behavioral) against the Roma minority in Romania, our study provides a possible starting point for future research aiming to reduce prejudice against this group.

This study has several limitations. Our sample was gender imbalanced, as the majority of our participants were females. We tested the influence of only one incidental emotion, i.e., disgust, which was found to be the emotion most commonly associated with the Roma minority (Petrík et al., 2021; Creţan et al., 2022). Nevertheless, previous studies have also found that other emotions can also increase prejudice. For example, in a Polish sample incidental anger also amplified the negative attitude toward the Romani minorities (Bukowski et al., 2014). As such, future studies should explore the influence of other incidental emotions such as fear or anger. Another important avenue of research is related to the possible effects of emotional regulation strategies. For example, participants’ habitual use of suppression or reappraisal, or their individual sensitivity to disgust could moderate the effects of the emotional induction tasks on the actual emotion that they experience, which could presumably determine prejudicial tendencies of varying magnitude.

In sum, our findings suggest that disgust increases prejudice, but only towards ethnic groups that are commonly associated with the same emotion. Inducing incidental disgust increased prejudice towards the Roma community while having no influence on the prejudice towards the individuals from the Hungarian minority, and the intensity of experienced disgust mediated the aforementioned relation. Moreover, the effect was observed on all three aspects of the prejudice: cognitive, affective, and behavioral. Our results offer support for the thesis of the specificity of the spill-over effect and may provide important information for future prejudice reduction interventions.

Interventions aiming at reducing negative bias towards minorities are usually based on general approaches that target the development of more favorable attitudes and inclusive behaviors. For example, such a general bias-reducing strategy is creating real or imagined contact between the participants and the discriminated group (Paolini et al., 2021). This strategy has been shown, in some cases, to reduce perceived differences between the majority and the discriminate group (Pettigrew and Tropp, 2008), that in turn creates the necessary premises for increased empathy (Pascal, 2020). However, there are also studies showing that these general approaches have limited effects in reducing prejudice, especially towards minorities such as the Roma ethnics (Pascal, 2020). This paper provides empirical support for the specificity of the spill-over effect which seems to be a possible explanation of the limited results of previous interventions. The main findings of our research suggest that future bias-reducing strategies could be more successful if they employed a tailored approach, targeting the specific emotion triggered by each discriminated group. For example, interventions aiming to reduce bias towards homosexual or Roma minorities might be more effective if they employed strategies that target the reduction of the negative emotion commonly associated with them, e.g., disgust (Roman et al., 2013; Kiss et al., 2020). Whereas, diminishing bias towards Arabic minorities, for example, could be more effective if they targeted emotions such as anger which are usually associated with them (Paolini et al., 2021). Future studies should empirically test the efficacy of more tailored prejudice reduction approaches based on the specific emotion elicited by the discriminated group.

## Data availability statement

The raw data supporting the conclusions of this article will be made available by the authors, without undue reservation.

## Ethics statement

The studies involving human participants were reviewed and approved by Comisia de etică a cercetării din cadrul Facultăţii de Psihologie şi Ştiinţe ale Educaţiei a Universităţii “Alexandru Ioan Cuza” Iaşi. The patients/participants provided their written informed consent to participate in this study.

## Author contributions

EP, AH, and FM contributed to the conception and design of the study and wrote sections of the manuscript. FM collected the data and organized the database. AH performed the statistical analysis and wrote the *results* section of the article. EP and FM wrote the first draft of the manuscript. EP and AH contributed to the manuscript revision and editing. All authors contributed to the article and approved the submitted version.

## Funding

This paper has been financed by the “Alexandru Ioan Cuza” University of Iasi, Romania, within the Project “The effects of perceived similarity on moral judgments. Cultural differences and mediating factors,” grant number GI-UAIC-2020-02.

## Conflict of interest

The authors declare that the research was conducted in the absence of any commercial or financial relationships that could be construed as a potential conflict of interest.

The reviewer EF declared a past co-authorship with the author AH to the handling editor.

## Publisher’s note

All claims expressed in this article are solely those of the authors and do not necessarily represent those of their affiliated organizations, or those of the publisher, the editors and the reviewers. Any product that may be evaluated in this article, or claim that may be made by its manufacturer, is not guaranteed or endorsed by the publisher.
